# Cytotoxicity and Antimicrobial Effects of a New Fast-Set MTA

**DOI:** 10.1155/2017/2071247

**Published:** 2017-02-20

**Authors:** Michelle Shin, Jung-Wei Chen, Chi-Yang Tsai, Raydolfo Aprecio, Wu Zhang, Ji Min Yochim, Naichia Teng, Mahmoud Torabinejad

**Affiliations:** ^1^Department of Pediatric Dentistry, School of Dentistry, Loma Linda University, Loma Linda, CA 92350, USA; ^2^School of Dentistry, College of Oral Medicine, Taipei Medical University, 250 Wu-Hsing Street, Taipei, Taiwan; ^3^Biomechanics Research Laboratory, Center for Dental Research, Orthodontics and Dentofacial Orthopedics, Loma Linda University, Loma Linda, CA 92350, USA; ^4^Dental Education Services, School of Dentistry, Loma Linda University, Loma Linda, CA 92350, USA; ^5^Division of Oral Rehabilitation and Center of Pediatric Dentistry, Department of Dentistry, Taipei Medical University Hospital, 252 Wu-Hsing Street, Taipei, Taiwan; ^6^Department of Endodontics, School of Dentistry, Loma Linda University, Loma Linda, CA 92350, USA

## Abstract

*Purpose*. To compare the biocompatibility and antimicrobial effectiveness of the new Fast-Set MTA (FS-MTA) with ProRoot MTA (RS-MTA).* Methods*. The agar overlay method with neutral red dye was used. L929 mouse fibroblast cells were cultured. The liquid and oil extracts and solid test material were placed on the agar overlay, four samples for each material. Phenol was used as the positive control and cottonseed oil and MEM extracts were used as negative controls. Cytotoxicity was examined by measuring the zones of decolorization and evaluating cell lysis under an inverted microscope using the established criteria after 24 and 48 hours. The antimicrobial test was performed using the Kirby-Bauer disk-diffusion method against* S. mutans*,* E. faecalis*,* F. nucleatum*,* P. gingivalis*, and* P. intermedia*. The size of the zone of inhibition was measured in millimeters.* Results.* There was no zone of decolorization seen under or around the test materials for FS-MTA and RS-MTA at 24 and 48 hours. The antimicrobial test demonstrated no inhibitory effect of FS-MTA or RS-MTA on any bacterial species after 24 and 48 hours.* Conclusions.* There was no cytotoxicity or bacterial inhibition observed by the new Fast-Set MTA when compared to the ProRoot MTA after setting.

## 1. Introduction

Mineral trioxide aggregate was primarily created as a root-end filling material in surgical endodontic procedures [1]. It has since then been indicated for other uses such as pulp capping, apexogenesis, and apexification in immature teeth with necrotic pulp, filling of root canals, treatment of horizontal root fractures, internal and external resorption, and repair of perforations [2]. It has been recognized as a bioactive material that is hard tissue conductive, hard tissue inductive, and biocompatible, which further idealizes MTA as a repair material in endodontic procedures [3]. In primary teeth, MTA is mainly used for direct pulp capping and pulpotomy procedures [2]. A new “Fast-Set MTA” has been developed by Dr. Mahmoud Torabinejad in Loma Linda, California.

Fast-Set MTA (FS-MTA) is a brand new material that was developed to be as effective as MTA with the added advantage of a quicker setting time. The setting time of the modified MTA has been reduced to 20 minutes. Current research studies are being conducted on bacterial microleakage and physical and chemical properties. Different methods have been tested to shorten the setting time of MTA, including a light-cured MTA and the addition of accelerants, such as disodium hydrogen orthophosphate and calcium lactate gluconate; all of these affect the physical or chemical properties of MTA in some way [4–6]. A fast-setting MTA will have the clinical advantages of increasing the usage of MTA in a dental practitioner's scope of practice, including pediatric dentistry. Because pediatric patients can often be restless and uncooperative, a fast-setting MTA can shorten the amount of chair time and increase the likelihood of a proper seal in a shorter amount of time. Since it is to be in permanent and close contact with periradicular tissues, it is important to assess its possible cytotoxic effects on living cells [7]. Bacteria are the main culprits for the development of pulp and periapical disease; since existing materials may not provide a perfect and hermetic seal, it is desirable that the material can prevent bacterial growth [8].

The purpose of this study is to compare the biocompatibility and antimicrobial effectiveness in vitro of the new gray Fast-Set MTA (FS-MTA) with regular ProRoot Gray MTA (RS-MTA) by using two tests: the agar diffusion test for cytotoxicity on L929 mouse fibroblast cells and the Kirby-Bauer disk-diffusion method for measuring the antimicrobial effect.

## 2. Materials and Methods

### 2.1. Test Material Preparation

#### 2.1.1. Solid Material

The gray ProRoot MTA (Dentsply, Lot Number 12120401B) was mixed according to the manufacturer's instructions and condensed into an internal diameter of 10 mm and thickness of 2 mm Teflon o-rings, which were then allowed to completely set in an incubator at 37°C for 24 hours. For the test material, a L/P = 1 : 4 ratio of FS-MTA was mixed and condensed into the o-rings and allowed to set in the same conditions. It was determined that the material was completely set when the tip of a clean explorer did not leave an indentation in the cement with typical force.

#### 2.1.2. Extracts

The test material was prepared in the same manner as above and then the sets of FS-MTA and RS-MTA were put in sterile water prepared at concentrations of 0.2 g/mL to determine the volume of the solvent for the liquid extract. Eagle's minimal essential medium (MEM) or PBS (FS-MTA MEM/PBS and RS-MTA MEM/PBS) was used as the polar solvent, and cottonseed oil (FS-MTA oil and RS-MTA oil) was used as the nonpolar solvent. The extracts were incubated at 37°C in a humidified 5% CO_2_ incubator for 72 hours before the experiment. The extracts were filtered before use using a 0.22 *μ*m syringe filter on the day of the experiment.

### 2.2. Agar Overlay Method for Cytotoxicity

The cytotoxicity-agar diffusion test is a means to evaluate the cytotoxicity of a test material using the agar diffusion method as specified in ISO 7405 (2008) and ISO 10993-5 (2009) and adapted from the method used by Torabinejad et al. [9–11].

Mouse fibroblast L929 cells (NCTC clone 929, ATCC CCL 1, Manassas, VA) were grown to confluence and trypsinized using Trypsin-EDTA mixture (Difco Laboratories, Detroit, MI). The cell density was determined using an automated cell counter (Countess, Invitrogen, CA) and the concentration was adjusted to 1.0 × 10^5^ cells/mL. The cell suspensions were aliquoted into 6-well plates (5 mL/well) and incubated for 24 hours. The media were then withdrawn and an overlay agar (3% agar (Difco Laboratories, Detroit, MI) in 2x complete media at the ratio of 1 : 1), maintained at 45°C, was poured over the cell monolayer. The agar media were allowed to solidify at room temperature for 10 minutes. Then 200 *μ*L of neutral red solution (0.033%) was pipetted on the agar surface and the excess dye was removed after 20 minutes. The extract samples (50 *μ*L) were aliquoted onto sterile filter disks (6 mm diameter, AP Prefilter Filter Paper, Lot Number H8KM39502, Millipore Corporation, Bedford, MA). The filter disks and solid samples were placed at the center of the agar surfaces. The positive control used was phenol, and the negative controls were sterile MEM and cottonseed oil. The tests were run with four samples of each group, each in a separate 6-well plate to avoid cross contamination of the materials. The plates were incubated at 37°C in a humidified atmosphere of 5% CO_2_ for 24 and 48 hours. The cytotoxicity was examined by measuring the zone of decolorization and evaluating cell lysis under an inverted microscope using the established criteria (ISO 7405, 2008) after 24 and 48 hours of incubation.

### 2.3. Kirby-Bauer Disk-Diffusion Method for Antimicrobial Effect

The Kirby-Bauer disk-diffusion measures the effect of an antimicrobial agent against bacteria. The bacterial cultures used in this study were* Streptococcus mutans* (ATCC 25175),* Enterococcus faecalis* (ATCC 19433),* Fusobacterium nucleatum* (ATCC 49256),* Prevotella intermedia* (ATCC 49046), and* Porphyromonas gingivalis* (ATCC 33277). The bacteria density was adjusted to an optical density equivalent to 0.1 at 600 nm using the Ultrospec 10 Spectrophotometer (Amersham Biosciences). One hundred microliters of the adjusted concentration of bacterial culture was spread uniformly across the culture plate using an L-shaped glass rod. Trypticase Soy Agar (Becton Dickinson, Sparks, MD) was used to plate the* S. mutans* and* E. faecalis*. Brucella Blood Agar (BRU) Plates (Anaerobe Systems, Morgan Hill, CA) were used to plate* P. gingivalis, F. nucleatum,* and* P intermedia*. Four filter-paper disks (0.25 inches in diameter) were then placed on each quadrant on the surface of the agar plate, and 20 *μ*L of the test material extract was pipetted onto each of the filter-paper disks. The same procedure was applied for the negative control, phosphate buffered saline, and positive control, 5.25% sodium hypochlorite (NaOCl). NaOCl is the main irrigating solution used to dissolve organic matter and kill microbes effectively, and a higher concentration has a better effect than 1-2% solutions [12]. The solid samples of test materials were directly placed in contact to the surface of the agar. Each plate contained 4 samples of the test material. The data was collected by measuring the zone of inhibition in millimeters at 24 hours and 48 hours.

All the data was collected and tabulated for descriptive statistics. All the data collection was negative; thus, no inferential statistics were performed.

## 3. Results

**Figure 1:**
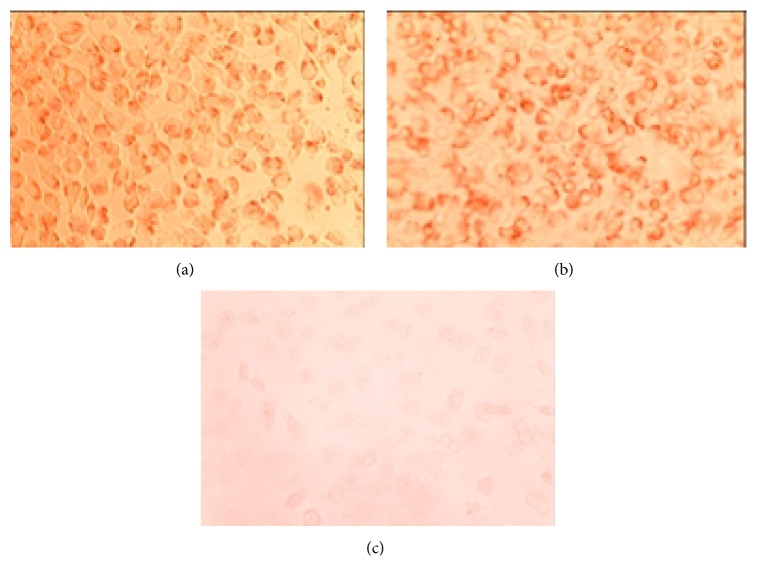
(a) FS-MTA oil sample at the border of the filter paper, (b) negative control, MEM, and (c) positive control, phenol. The cells are intact and in monolayer, with uptake of neutral red dye, indicating vitality of the cells, 100x.

For the cytotoxicity test, there was no zone of decolorization seen under or around the test materials for either the RS-MTA or the FS-MTA. The negative control did not show any zone of decolorization under or around the filter-paper disks at 24 and 48 hours. The cells were viewed under 40x and 100x magnification. The positive control showed complete decolorization and cellular lysis of all the wells as seen in Table 1. The results are reported in the table as the average of the four samples. The data was classified into a five-point cytotoxicity grading system. The negative controls and the gray RS-MTA and gray FS-MTA all received a cytotoxicity grade of 0, and the positive control received the maximum grade of 5 for decolorization and lysis index and was graded a maximum of 3 for cytotoxicity interpretation (severely cytotoxic).  Figure 1 illustrates the cells at a higher magnification (magnification at 100x), in the presence of FS-MTA oil sample at the border of the filter paper, the negative control, and positive controls. The fibroblast cells' uptake of neutral red dye after 24 hours, the presence of red dye, and absence of lysed cells show that these cells are vital.

**Figure 2:**
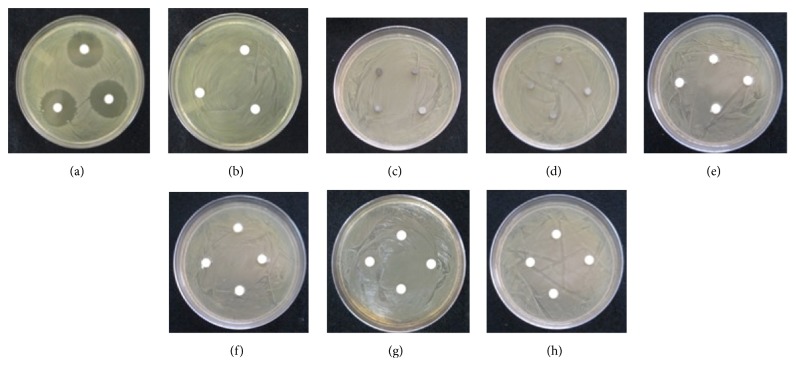
Agar diffusion test to measure the inhibition of FS-MTA and RS-MTA on bacterial growth; this particular grouping is result for* E. faecalis*. (a) Positive control, NaOCl 5.25%, (b) negative control, PBS, (c) RS-MTA solid, (d) FS-MTA solid, (e) RS-MTA oil, (f) FS-MTA oil, (g) RS-MTA PBS, and (h) FS-MTA PBS. Note that there is no zone of inhibition in any of the samples of RS-MTA or FS-MTA.

There was no inhibitory effect of FS-MTA or RS-MTA on the aerobic bacteria,* S. mutans* and* E. faecalis*, or the anaerobic bacteria,* F. nucleatum*,* P. intermedia*, or* P. gingivalis,* in 24 and 48 hours. The negative control did not show any zone of inhibition in all of the bacteria species. The positive control showed zone of inhibition in all the bacteria species (Table 2). The results are reported as the average of the three samples. Figures 2(a)–2(h) show the results of FS-MTA and RS-MTA on* E. faecalis* when compared to the control groups; no zone of inhibition was detected.

## 4. Discussion

Multiple tests to determine the biocompatibility of dental materials exist, such as cytotoxicity tests in tissue cultures, in vivo subcutaneous or bone implant tests, and usage tests [11]. Cytotoxicity tests are inexpensive, simple, and rapid and can be used as a screening test, which can provide helpful information as to whether or not a material should be further tested for potential use in humans. The types of cell lines that are used for tissue culture cytotoxicity tests include L929 mouse fibroblasts, gingival fibroblast cells, and human PDL cells [13–15]. This study used L929 mouse fibroblasts, as it is a commonly used cell line.

There are three qualitative cytotoxicity tests that are commonly used for testing medical materials: the direct contact procedure, agar diffusion assay, and MEM elution assay. The direct contact procedure is recommended for 4 low-density materials, agar diffusion assay is appropriate for high-density materials, and the MEM elution assay uses different extracting media and extraction conditions to test devices according to the actual conditions or to exaggerate those conditions [16]. A zone of malformed, degenerative, or lysed cells under and around the test material shows that the material is cytotoxic. Our test results did not show any malformed or degenerated cells under or around the samples of FS-MTA, in either the extracts or the solid samples.

The agar overlay method has been used in multiple cytotoxicity tests, including Torabinejad et al. [11], who reported a zone of lysis around samples of fresh and set gray MTA. Haglund reported there were denatured medium proteins and dead cells adjacent to the material, but only in the fresh MTA group; however the set MTA had no effect on cell morphology [17]. Miranda et al. tested 48-hour set MTA, which showed viable cells around the pellets, and dead cells were observed only under the material [18]. The results of our study showed that there was no effect on cell morphology from either the FS-MTA or RS-MTA in the set form or the extract forms under or around the test material or extract after 24 and 48 hours.

MTA has been shown to be one of the least cytotoxic dental materials in comparison to Super EBA, IRM, amalgam, various types of glass ionomers, gutta-percha, and Dycal [13]. Our study showed that this modified form of FS-MTA does not show any cytotoxic effects on L929 mouse fibroblast cells. Although our tests were sufficient enough to screen this new material for cytotoxicity, biocompatibility testing regulation (ANSI/AAMI/ISO 10993-5:2009) has stated that qualitative tests are appropriate for screening purpose but quantitative evaluation would be preferable [16]. Our study demonstrated that the new FS-MTA does not have any cytotoxic properties and is comparable to the RS-MTA.

The Kirby-Bauer disk-diffusion method was the test used to evaluate the antibacterial properties of FS-MTA in comparison to RS-MTA. This is one of the most widely used in vitro methods for the evaluation of antimicrobial activity and allows direct comparisons between materials that could have antibacterial action [19, 20]. It has been shown that the antibacterial effect of sealers generally decreases in a set state, because once the setting reaction has been completed, diffusion in the agar is difficult [19]; the same could be stated of MTA, which is cement that undergoes a setting reaction. For this reason, we used extracts of the test materials to ensure that the leachable elements were evaluated as well as the solid material.

We used two facultative bacteria species and three anaerobic bacteria species for our tests. Of more than 300 bacterial species that are present in the normal oral flora, a relatively small group colonizes infected root canals—mainly of strict anaerobes and some facultative anaerobes and usually no aerobes [21]. Our study did not show any inhibitory effect of the FS-MTA or RS-MTA on the facultative anaerobic species* S. mutans* and* E. faecalis*, nor was there any inhibitory effect on the anaerobic bacteria,* F. nucleatum*,* P. gingivalis*, and* P. intermedia*.

*S. mutans* has been found to be present in the dentinal tubules of 48.7% of infected root canals and is the primary causal agent and pathogenic species responsible for dental caries because of its ability to produce acid and initiate the caries process [22]. In infected primary teeth,* P. gingivalis, P. intermedia*, and* F. nucleatum* were found in high percentages in both the pulp chamber and root canals [23].* E. faecalis* is a primary pathogenic factor in endodontic treatment and is detectable in about 77% of cases that are resistant to treatment [24]. Tanomaru-Filho et al. showed that gray ProRoot MTA inhibited various facultative bacteria in a freshly mixed state [25].

Torabinejad et al. reported that both fresh and set MTA had antibacterial effect on* S. mitis* but not* S. faecalis, S. aureus*, and* B. subtilis*, all of which are facultative bacteria [21]. The fresh and set MTA also showed some antibacterial effect on* S. mutans*. Of the anaerobic bacteria, the same study showed that there was no antibacterial effect against any of the anaerobic bacteria tested:* P. buccae, B. fragilis, P. intermedia, P. melaninogenica*,* P. anaerobius, F. necrophorum*, and* F. nucleatum* [21]. Heyder et al. discovered that ProRoot MTA only had an antibacterial effect in a freshly mixed state but did not inhibit any growth on anaerobes. ProRoot MTA did not have any inhibitory effect on* E. faecalis* in either the freshly mixed or set forms [24]. However, in our study, we did not see any inhibition of bacterial growth in the extracts or solid samples of the FS-MTA and RS-MTA.

The antimicrobial effects seen in MTA are thought to be from its high pH or release of diffusable substances into the growth medium, especially in the freshly mixed state [3]. We did not use freshly mixed FS-MTA and RS-MTA in our study, which may have shown a different outcome; however, we did use extracts that should contain any leachable components of the FS-MTA and RS-MTA if they were indeed present [16]. The pH of the extracts was not tested before the placement of the disks onto the bacteria-inoculated agar plates. It may be helpful to test the new material, FS-MTA, in a freshly mixed state to evaluate the antibacterial effect it may have before complete setting of the cement. Ultimately, our study showed that there was no difference between the antibacterial properties between the new FS-MTA and RS-MTA, as there was no inhibition of the bacterial species tested.

To improve this study, the biocompatibility of the new FS-MTA can be tested quantitatively with a test such as the MTT assay and can further be tested on human gingival fibroblast cells rather than mouse fibroblast cells. To further test the antibacterial properties of the new FS-MTA, it may be helpful to compare the freshly mixed state of the new product with the RS-MTA.

## 5. Conclusion

Under the condition of the present study, the new FS-MTA was not cytotoxic in the L929 mouse fibroblast cell line, and there was no difference between the FS-MTA and the RS-MTA. Also, the new FS-MTA did not show antimicrobial properties against the facultative anaerobic species,* S. mutans* and* E. faecalis*, or the strict anaerobic species,* P. gingivalis, P. intermedia*, and* F. nucleatum*. There was no difference in antimicrobial effect between the FS-MTA and the RS-MTA.

## Figures and Tables

**Table 1:** Cytotoxicity evaluation of FS-MTA and RS-MTA, using evaluation criteria for agar diffusion test (ISO 7405, 2008). For zone index, 0 indicates no detectable decolorization zone; a score of 5 indicates a zone involving the entire dish. For lysis index, a score of 0 indicates no observable cytotoxicity, 5 indicates > 80% of the decolorized zone affected. For interpretation of cytotoxicity, score of 0 indicates being noncytotoxic, and 3 indicates severe toxicity.

Material	24 hours			48 hours		
	Zone index	Lysis index	Interpretation	Zone index	Lysis index	Interpretation
MEM (−control)	0	0	Noncytotoxic	0	0	Noncytotoxic
Cottonseed oil (−control)	0	0	Noncytotoxic	0	0	Noncytotoxic
Phenol (+control)	5	5	Severely cytotoxic	5	5	Severely cytotoxic
RS-MTA solid	0	0	Noncytotoxic	0	0	Noncytotoxic
RS-MTA MEM	0	0	Noncytotoxic	0	0	Noncytotoxic
RS-MTA oil	0	0	Noncytotoxic	0	0	Noncytotoxic
FS-MTA solid	0	0	Noncytotoxic	0	0	Noncytotoxic
FS-MTA MEM	0	0	Noncytotoxic	0	0	Noncytotoxic
FS-MTA oil	0	0	Noncytotoxic	0	0	Noncytotoxic

**Table 2:** Measurements of zone of inhibition in millimeters (mm) at 24 and 48 hours for RS-MTA and FS-MTA solid, extract, and oil samples at 24 and 48 hours on bacterial species, and *N* = 4.

Material	*S. mutans*		*E. faecalis*		*P. gingivalis*		*P. intermedia*		*F. nucleatum*	
	24 h	48 h	24 h	48 h	24 h	48 h	24 h	48 h	24 h	48 h
PBS (−control)	0.00	0.00	0.00	0.00	0.00	0.00	0.00	0.00	0.00	0.00
NaOCl 5.25% (+control)	29.17 ± 0.29	29.17 ± 0.29	25.67 ± 2.31	25.67 ± 2.31	28.33 ± 2.47	28.33 ± 2.47	9.67 ± 1.15	9.67 ± 1.15	10.00 ± 0.50	10.00 ± 0.50
RS-MTA PBS	0.00	0.00	0.00	0.00	0.00	0.00	0.00	0.00	0.00	0.00
RS-MTA oil	0.00	0.00	0.00	0.00	0.00	0.00	0.00	0.00	0.00	0.00
RS-MTA solid	0.00	0.00	0.00	0.00	0.00	0.00	0.00	0.00	0.00	0.00
FS-MTA PBS	0.00	0.00	0.00	0.00	0.00	0.00	0.00	0.00	0.00	0.00
FS-MTA oil	0.00	0.00	0.00	0.00	0.00	0.00	0.00	0.00	0.00	0.00
FS-MTA solid	0.00	0.00	0.00	0.00	0.00	0.00	0.00	0.00	0.00	0.00
